# Discrimination Against Women in Sport: A Scopus-Based Bibliometric Analysis (1995–2026)

**DOI:** 10.3390/bs16050753

**Published:** 2026-05-12

**Authors:** Vinu Wilson, Dilshit Azeezul Kabeer, Josyula Tejaswi, Ashif Ali Narippatta Kappoor, Jayaraman Sundararaja, Jolita Vveinhardt, Karuppasamy Govindasamy

**Affiliations:** 1 Department of Physical Education & Sports, Pondicherry University, Puducherry 605014, India; vinu@pondiuni.ac.in (V.W.); dilshith.dpe@pondiuni.ac.in (D.A.K.); ashifalink4@pondiuni.ac.in (A.A.N.K.); 2 Symbiosis School of Sports Sciences, Symbiosis International (Deemed University), Gram: Lavale, Tal: Mulshi, Pune 412115, Maharashtra, India; tejaswi.josyula@ssss.edu.in; 3 Department of Physical Education and Sports, Central University of Tamil Nadu, Thiruvarur 610005, Tamil Nadu, India; jayaraman@cutn.ac.in; 4 Institute of Sport Science and Innovations, Lithuanian Sports University, Kaunas 44221, Lithuania; 5 Department of Sports, Recreation and Wellness, Symbiosis International (Deemed University), Hyderabad Campus, Modallaguda (V), Nandigama (M), Rangareddy 509217, Telangana, India

**Keywords:** women sport, gender discrimination, sexism, harassment, leadership, bibliometric analysis

## Abstract

Background: Gender discrimination in sport remains a persistent global issue, reflected in women’s limited participation, leadership representation, media visibility, salary equity, and personal safety. These forms of discrimination also negatively affect athletes’ psychological well-being, mental health, and overall sports experience. Despite growing scholarly attention over the past three decades, a comprehensive quantitative synthesis of this research area has been lacking. Methodology: A bibliometric analysis of 397 peer-reviewed documents published between 1995 and 2026 was conducted using the Scopus database. Data were analysed through the Bibliometric R package 4.2.1 and Biblioshiny interface. Science-mapping techniques including keyword co-occurrence, thematic clustering, thematic evolution, and collaboration network analysis were combined with performance indicators such as annual publication output, leading sources, author productivity, and citation impact. Results: Scientific production increased markedly after the mid-2010s, involving 187 sources and 1106 authors, with rising collaboration and citation influence. Core research themes included gender inequality, leadership exclusion, media representation, harassment and abuse, and structural discrimination in sports systems. Importantly, many of these themes are directly linked to reduced athlete well-being, including increased stress, anxiety, and decreased participation. Recent thematic developments highlighted intersectionality, safeguarding, inclusion, governance, and athlete welfare. Conclusion: Research on discrimination against women in sport has evolved into a multidisciplinary, policy-relevant field. Addressing gender discrimination is essential not only to achieving equity but also to improving athletes’ subjective well-being and long-term participation in sport. However, significant gaps remain, particularly in Global South contexts and intervention-based studies, indicating the need for stronger evidence-driven strategies to advance gender equity, inclusion, and ethical governance in sport.

## 1. Introduction

Sport is a salient social institution that reflects and reproduces the power structures in society, including gender structures embedded in popular culture and economic systems (Coakley, 2017; Hargreaves, 2002; Messner, 2002). Historically, modern sport has been organised in ways that reflect masculine ideals of competition, leadership, and embodiment, thereby excluding women and limiting their opportunities, sources, and recognition (Claringbould & Knoppers, 2012; A. H. Shaw et al., 2023). Despite significant improvements in women’s participation rates worldwide, gender-based discrimination persists across various aspects of sport, including access to facilities and funding, roles in leadership and decision making, media coverage, pay and compensation, and protective measures (Burton, 2015; Fink, 2015). Importantly, these forms of discrimination are not only structural concerns but also have direct implications for athletes’ subjective well-being, including their mental health, motivation, sense of belonging, and overall sports experience.

While these disparities are often described in terms of inequality, it is important to distinguish this from the concept of discrimination. Inequality broadly denotes uneven distribution of opportunities and outcomes, whereas discrimination refers to the systematic and often institutionalised processes of differential treatment based on gender that actively produce and sustain such inequalities (Atkinson, 2015). In sports contexts, many observed inequalities, such as underrepresentation in leadership, limited media visibility, and unequal remuneration, are not merely incidental but are rooted in entrenched structural, cultural, and organisational practices. These include gender bias in recruitment and promotion, exclusionary governance mechanisms, stereotypical media portrayals, and experiences of harassment and abuse (Kerr et al., 2019).

Therefore, the concept of discrimination provides a more critical and explanatory lens, enabling the analysis of both overt and subtle mechanisms through which gendered power relations are reproduced within sports systems. Contemporary scholarship has increasingly shifted from descriptive accounts of inequality toward more critical and intersectional examinations of discrimination, focusing on issues such as safeguarding, institutional accountability, and athlete welfare (Fink, 2016). This conceptual orientation allows for a deeper understanding of how structural barriers operate and why gender disparities persist despite policy-level efforts toward equality (Fink, 2016; Leeds & Leeds, 2013).

Female athletes in professional sports such as football (soccer) and basketball continue to experience lower levels of job security, remuneration, and contractual stability compared with their male counterparts, despite comparable international performance success; although recent developments in countries such as the United States, Norway, and Australia have introduced equal pay frameworks in select contexts, broader commercial disparities driven by historical underinvestment, limited media coverage, and sponsorship inequalities continue to constrain the overall economic valuation of women’s sport (Carrick, 2024; Cooky & McDonald, 2005; Culvin & Bowes, 2021). The presence of gendered power imbalance is also distinctly seen in sports governance, with the underrepresentation of females in the field of coaching, refereeing, and executive leadership being notably higher, a situation that is explained by structural, informal, and glass ceiling effects (LaVoi & Dutove, 2012; Piggott & Pike, 2020). These inequalities within an organisation not only limit the career paths of women but also influence policy priorities, resource allocation, and institutional responses to discrimination.

Media representation represents another topical area of gender discrimination in sports here. The ongoing marginalisation, sexualisation, and trivialisation of women athletes in both traditional and online media is a process documented by a large body of academic literature, thus supporting symbolic hierarchies that ideally make the sport of men the norm (Bruce, 2016; Cooky et al., 2021; Toffoletti & Thorpe, 2018). These representational practices define how people think about athletic legitimacy, commercial worthiness, and cultural relevance and continue to reinforce material inequalities in funding, sponsorship, and visibility (Antunovic & Hardin, 2015). Academic interest and policy debates have increasingly focused on the topic of harassment, abuse and gender-based violence in sport over the past few years (Mountjoy et al., 2016). Cases of high profile, including elite, youth, and community sports systems, have highlighted systemic failures in protection and reporting infrastructures, and institutional responsibility which have disproportionately affected women and girls. These issues overlap with broader questions about the welfare of athletes, human rights, and ethical governance, thereby establishing gender discrimination in sport as a social-justice and public-health challenge rather than a participation concern (Bekker & Posbergh, 2022). Such experiences of discrimination, harassment, and exclusion are strongly associated with negative well-being outcomes, including psychological distress, anxiety, reduced self-esteem, and withdrawal from sports participation. Therefore, gender discrimination in sport must also be understood as a critical determinant of athlete well-being.

Academic interest in discrimination against women in sport has grown significantly over the last 30 years. The first studies mainly reported gender disparities in participation and access. Still, later research has employed critical and feminist approaches to examine organisational culture, policy frameworks, leadership inequality, and power relationships in media (Burton & Leberman, 2017; Messner, 2002). Intersectional approaches are becoming increasingly popular in recent scholarship, highlighting the role of gender discrimination in sport as conditioned by intersecting identities, including race, ethnicity, class, disability, sexuality, and nationality (Carter-Francique & Flowers, 2013; Ratna, 2011). The literature has thus become very interdisciplinary, encompassing sports sociology, gender studies, psychology, management, public policy, and law. Despite the important insights this growing body of research has produced, its expansion and conceptual heterogeneity make it challenging to use traditional narrative or systematic reviews to identify prevalent themes, contributing factors, patterns of collaboration, and research gaps (Pritchard, 1969; Zupic & Čater, 2015). Bibliometric analysis is a robust, objective, and repeatable technique of mapping the intellectual landscape and development of a research discipline through quantitative analysis of publication patterns, citation influence, authorship patterns, and the patterns of co-occurring keywords (Aria & Cuccurullo, 2017; Donthu et al., 2021; Velez-Estevez et al., 2023). These analyses have become widely used in research across both sport and the social sciences to integrate large, disaggregated studies and to serve as the basis for future research agendas.

Thus, the current research study aims to conduct an extensive bibliometric review of Scopus sources on discrimination against women in sport published between 1995 and 2026. To be more precise, this paper aims to: (i) determine the temporal dynamics and pattern of scientific output; (ii) define the most popular journals, authors, institutions, and countries; (iii) chart the patterns of collaboration and intellectual networks within the discipline; and (iv) reveal prevalent thematic patterns and uncharted research gaps. This study offers an evidence-based contribution to knowledge-based scholarship, policy making, and governance reforms that would support the promotion of gender equity in sport. In addition, by mapping the evolution of research on gender discrimination, this study also contributes to understanding its implications for athletes’ subjective well-being, aligning with the broader objective of promoting healthier, more inclusive sports environments.

## 2. Materials and Methods

### 2.1. Research Design

This study adopts a bibliometric research design to systematically map and analyse the scientific literature on discrimination against women in sport. Bibliometric analysis is a quantitative and reproducible approach that enables the identification of publication trends, influential sources, collaboration patterns, and thematic structures within a research field. To enhance transparency and methodological rigour, this study followed structured review procedures aligned with established guidelines such as PRISMA (Preferred Reporting Items for Systematic Reviews and Meta-Analyses) for literature identification, screening, and inclusion. The overall research process involved database selection, search strategy development, data extraction, data cleaning, and bibliometric analysis using specialised tools.

### 2.2. Data Source and Search Strategy

The bibliometric data used to answer this question were obtained from the Scopus database, an information system widely recognised for covering the peer-reviewed literature in the fields of sports sciences, social sciences, humanities, and management. The selection of Scopus was driven by the standardisation of its indexation protocols, comprehensive citation metadata, and its suitability for bibliometric and science-mapping studies. A search plan was designed using the TITLE-ABS-KEY field, which helped select publications that directly address the discrimination against women in sport. The search query included lexical terms related to women and female athletes, as well as sport-related situations and discrimination-related constructs, such as gender inequality, sexism, harassment, leadership obstacles, exclusion, and abuse. To map the field’s longitudinal path, the query was limited to publications from 1995 to 2026. The last search was conducted in January 2026, and all retrieved records were exported to CSV format. The exported data included extensive bibliographic information, including titles, abstracts, author affiliations, indexed keywords, cited references, publication years, and the number of citations. A brief overview of the Scopus search query that was developed through the use of Boolean operators in the TITLE-ABS-KEY field, which was aimed at retrieving relevant studies on discrimination against women in sport over the years 1995–2026, is given in (Table 1).

### 2.3. Eligibility Criteria and Study Selection

**Figure 1:**
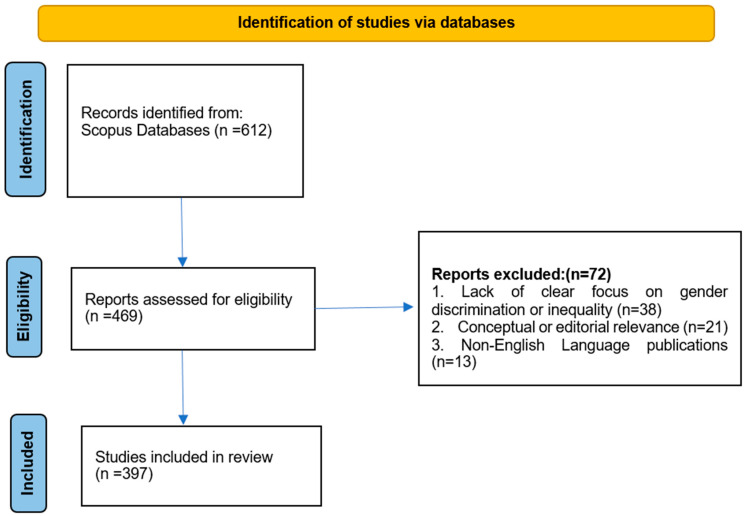
PRISMA flow diagram illustrating the process of study selection for the bibliometric analysis. It depicts the stages of identification, screening, eligibility assessment, and final inclusion of studies retrieved from the Scopus database, focusing on discrimination against women in sport over the period 1995–2026.

To ensure conceptual and disciplinary relevance, the included studies were drawn from interdisciplinary fields indexed in Scopus, including sports sciences, physical education, sports management, sociology, gender studies, psychology, and public policy. These disciplines were considered essential to capturing both performance-related and sociocultural dimensions of gender-based discrimination in sport. Studies from unrelated domains such as purely biomedical or clinical sciences without a sports context, engineering, or general management fields not addressing sports systems were excluded unless they explicitly examined gender discrimination within sports environments. We considered publications released between 1995 and 2026; studies explicitly addressing discrimination, inequality, bias, harassment, abuse, leadership exclusion, or structural barriers affecting women or female athletes within sports contexts; and English-language publications. The first search in the database returned 612 results, and all of them were retained after copying, using the built-in deduplication mechanism that detected and eliminated duplicates. Abstract screening resulted in the elimination of 143 records that failed to meet the inclusion criteria, as the majority were irrelevant to the context of sport and focused on gender discrimination without analysing it through the prism of sociocultural and structural factors. Four hundred six hundred and ninety-nine articles were then subjected to full-text evaluation, and 72 studies were eliminated by lack of relevance to the themes of discrimination, lack of conceptual or editorial content, or non-English language publication. After this stringent screening and eligibility procedure, 397 studies remained in the final dataset on which the bibliometric analysis was conducted. A bibliometric analysis PRISMA flow diagram is used to demonstrate this study’s selection process (Figure 1).

### 2.4. Bibliometric Analysis

Bibliometric analysis was employed as the primary analytical approach due to its ability to systematically evaluate large volumes of scholarly research and identify patterns in research productivity, intellectual structure, and thematic evolution (Donthu et al., 2021; Zupic & Čater, 2015). This method is particularly suitable for multidisciplinary domains such as gender discrimination in sport, where conceptual diversity and fragmented scholarship limit the effectiveness of traditional narrative or systematic reviews. Bibliometric techniques offer objective, reproducible, and data-driven insights into publication trends, citation impact, collaboration networks, and emerging research themes (Aria & Cuccurullo, 2017).

The analysis was conducted using Biblioshiny, the web-based interface of the Bibliometrix R (version 4.2.1) package, which facilitates comprehensive performance evaluation and science-mapping visualisation. Two complementary analytical frameworks were applied: performance analysis (PA) and science-mapping analysis (SMA) (Donthu et al., 2021). Performance analysis focused on descriptive indicators of the research landscape, including annual scientific production, leading publication sources, author productivity, collaboration patterns, and citation impact measured through total citations and average citations per document.

In contrast, science-mapping analysis examined the intellectual and conceptual structure of the field through keyword frequency and co-occurrence analysis, thematic clustering, thematic evolution, and author and country collaboration networks (Zupic & Čater, 2015). Prior to analysis, data preprocessing was performed by standardising author names, keywords, and institutional affiliations to minimise duplication and enhance network accuracy. Descriptive statistics were used to summarise the structural characteristics of the dataset, followed by thematic analysis to identify dominant and emerging research clusters.

Furthermore, Table 2 presents the key bibliometric indicators and analytical categories employed to assess both performance metrics and science-mapping structures within the dataset.

Bibliometric analysis (BA), initially proposed by Pritchard (1969), is a quantitative methodological approach used to determine influential scholars, trace the evolution of research fields, and identify emergent research trends, based on metrics derived from analysing the scholarly literature. This study followed the bibliometric workflow proposed in (Zupic & Čater, 2015) and analytical procedures adopted in recent bibliometric investigations, including (Eldos et al., 2025). The analytical process is divided into three main steps: the expression of the research purpose and identification of keywords; systematic data gathering and filtering; and subsequent data analysis and visualisation in the bibliometric analysis. Indicators based on publications and citations, including total publications, total citations, and fractionalised authorship, were used to identify dominant contributors. Additional analyses included citation analysis, co-citation analysis, bibliographic coupling, and co-word analysis, in which the authors’ keywords served as the primary units of analysis. Visualisation techniques recommended in (Aria & Cuccurullo, 2017), (Velez-Estevez et al., 2023), and (Donthu et al., 2021) were applied using Biblioshiny (version 4.2.1), Tableau (version 2023.1), and Java-based visualisation tools (version 1.6.20).

The assessment of metadata quality was done thoroughly through the Biblioshiny competence report. The abstract (AB), document type (DT), source title (SO), language (LA), publication year (PY), title (TI), and total citations (TC) variables were rated excellent, thus indicating high reliability. Author information (AU), affiliations (C1), cited references (CR), and DOI (DI) received a good rating. Keywords Author (DE) and author information (RP) were rated acceptable, while Keyword Plus (ID) and subject categories (WC) were rated poor, indicating limitations in thematic granularity. However, the aggregate data were deemed robust enough to undertake extensive bibliometric and science-mapping studies.

## 3. Results

This bibliometric analysis is presented in two major sections: Performance Analysis (PA) and Science-Mapping Analysis (SMA). The PA Section provides a quantitative evaluation of the academic environment regarding discrimination against women in sport, including patterns in annual scientific publications, the most popular publication outlets, the most frequently cited articles, and the most active and influential authors. Furthermore, PA examines the distribution of research output across nations, institutions, and funding bodies worldwide, thus providing information on the geographical and organisational clustering of knowledge production in this sphere.

The SMA part explores the intellectual and conceptual framework of the studies on discrimination against women in sport. In particular, it focuses on thematic concentration, thematic development over time, and new and potential future research avenues, thereby clarifying the shift in academic interest towards more critical, policy-focused, and intersectional approaches to early descriptive explanations.

### 3.1. Performance Analysis (PA)

**Figure 2:**
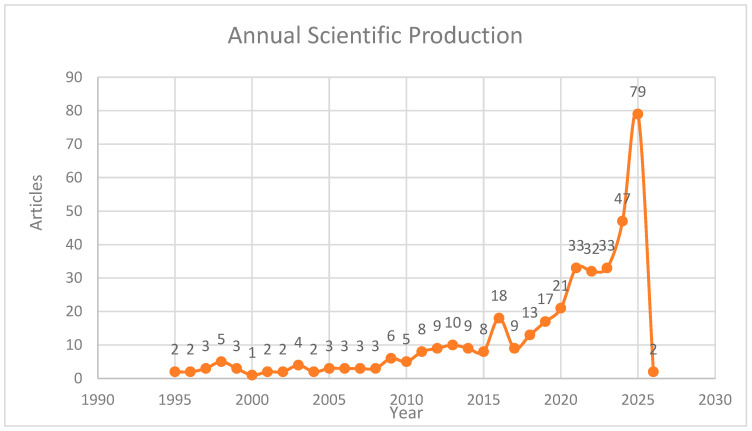
Annual scientific production.

The Performance Analysis part includes four principal evaluations of this paper on discrimination against women in sport: annual scientific production (Figure 2), the most prolific sources of research articles in terms of the number of publications (Table 3), the most influential works in terms of total citation numbers (Table 4), the most influential articles by decade on women’s discrimination in sport (Table 5), and the most prolific and influential authors in the sphere of discrimination against women in sport (Table 6). These thematic patterns provide important insights into the trajectory of research on discrimination against women in sport. The dominance of core terms such as gender, sport, and women reflects the foundational focus of the field on identifying and documenting inequalities within sports systems. However, the emergence of themes related to media representation, harassment, leadership, and inclusion indicates a clear shift toward more critical and multidimensional analyses of discrimination.

Furthermore, the increasing presence of keywords such as intersectionality, mental health, and social media suggests that the field is evolving toward more nuanced, interdisciplinary, and context-sensitive approaches. This progression aligns with this study’s objective of mapping the intellectual and thematic development of the field, demonstrating a movement from descriptive accounts toward policy-relevant, intersectional, and action-oriented research frameworks. These trends highlight the growing emphasis on athlete welfare, institutional accountability, and inclusive governance, thereby shaping future research directions in this domain.

### 3.2. Trends in Annual Scientific Production Annual Scientific Production

The scientific output on discrimination against women in sport, annually from 1995 to 2026, identifies the temporal distribution and the rate of increase in publications. The initial period of research work (1995–2008) is characterised by a low level of scholarly production, with the number of articles published each year typically ranging from one to five. Together, this time span accounted for less than 10 percent of the overall publications, indicating that gender-based discrimination in sport was originally a peripheral field of scholarly research, as shown in Figure 2.

Between 2009 and 2014, a transitional growth phase was observed, during which publication activity increased, accounting for about 15 to 18 percent of the entire literature. This stage is associated with the increased awareness of gender equity concerns in sport alongside the rise in women’s participation, the era of governance reforms, and a broadening policy debate on inclusion and equity in sports systems. The most noticeable increase in scientific output occurred in 2015 and onwards, which can be considered a critical point in scientific production. Articles published from 2015 to 2026 constitute more than 70 per cent of the overall research output, indicating a substantial focus on scholarly attention. The number of articles published annually rose sharply, and production grew by over 120 per cent between 2016 and 2020, reaching a high of 79 in 2024, which alone accounts for almost 20 per cent of the total data. This boom is linked to an increased international sensitivity to women’s rights in sport, greater attention to inequality in leadership, media bias, and failure in safeguarding, and the impact of global policy and advocacy groups.

The seemingly reduced number of publications in 2026 should be viewed with some reservations, as it is not a sign of an actual slowdown in research activity but rather a case of partial-year indexing. Comprehensively, the trend in the volume of annual scientific production indicates that research on discrimination against women in sport has shifted from a low-volume, peripheral subject to a fast-growing, well-established area of study. The continuous expansion over the past 10 years indicates the field’s growing use in sports sociology, management, policy, and governance, as well as its high potential for future scholarly growth.

### 3.3. Leading Sources of Publications

Table 3 presents the most common sources used in research on discrimination against women in sport, ranked by the number of publications. This discussion indicates that a relatively limited number of journals account for a high proportion of the overall production, thereby highlighting the monopolistic character of academic discourse in specialised outlets in sports sociology, sports management, and gender studies. These sources are at the centre of developing theoretical discussions, methodological theories, and policy-based discussions concerning gender discrimination in sport.

**Table 3:** Leading sources of publications on women’s discrimination in sport (1995–2026).

Rank	Source Title		No. (Publications)	SCY	CiteScore (2026)	SNIP (2020–2026)
1	*Communication and Sport*	Sage	17	2015–2026	3.8	1.5
2	*Women in Sport and Physical Activity Journal*	Human Kinetics	16	218–2026	3.8	1.17
3	*Frontiers in Psychology*	Frontiers	11	2010–2026	6.3	2.9
4	*Frontiers in Sports and Active Living*	Frontiers	11	2019–2026	3.8	0.86
5	*Journal of Sport and Social Issues*	Sage	11	1977–2026	3.2	1.47
6	*International Review for the Sociology of Sport*	Sage	10	1966–2026	5.2	1.84
7	*Sex Roles*	Springer	8	1975–2026	6.4	2.19
8	*Sport, Ethics and Philosophy*	Taylor & Francis	8	2007–2026	3.3	1.9
9	*European Journal for Sport and Society*	Taylor & Francis	7	2024–2026	5.4	2.6
10	*Journal of Physical Education and Sport*	Ro Sport & Art Association	7	2011–2025	3.2	0.5

### 3.4. Most Influential Articles

Table 4 presents the most highly cited articles in the field of discrimination against women in sport, serving to identify the intellectual foundations and most influential scholarly contributions shaping this research area. The purpose of this table is not only to highlight citation impact but also to indicate key themes, dominant research directions, and the evolution of scholarly focus within the field. It is important to note that the predominance of earlier publications among the most cited articles reflects a well-established bibliometric pattern, where foundational studies accumulate citations over time due to their longer presence in the literature. Many of these highly cited works have played a critical role in shaping the conceptual and theoretical foundations of research on discrimination against women in sport, particularly in areas such as gender representation, media discourse, and power relations.

**Table 4:** The most cited articles.

	Paper	DOI	TC	TC pY	N TC
1.	Heywood, L.L., 2003, Built to win: the female athlete as cult icon (Heywood & Dworkin, 2003).		276	11.5	2.09
2.	New Rules for New Times: Sportswomen and Media Representation in the Third Wave (Bruce, 2016).		257	23.36	8.51
3.	Intraindividual Variability of Coaching Behaviours and Athlete Motivation (Journal of Sport & Exercise Psychology)		212	7.85	1
4.	“He Owned Me Basically…” Women’s Experience of Sexual Abuse in Sport (Brackenridge, 1997) (International Review for the Sociology of Sport)		186	6.2	1.68
5.	Action Sports, Social Media, and New Technologies: Towards a Research Agenda (Thorpe, 2017).		167	16.7	5.62
6.	Television Viewers’ Perceptions of African American and White Athletic Bodies (Harrison & Fredrickson, 2003).		161	6.70	1.22
7.	Women, Sport, and Physicality: Preliminary Findings from a Canadian Study (Young, 1997).		149	4.65	1.89
8.	“I am Not Saying Women Can’t Coach”: A Qualitative Study of Gendered Coaching Discourses (European Journal for Sport and Society)		118	13.11	5.9
9.	Gender Stereotyping in Televised Media Sport Coverage (Koivula, 1999).		118	4.21	1.63
10.	Sex Objects, Athletes, and Sexy Athletes: How Media Representations of Women Athletes Can Impact Adolescent Girls (Daniels, 2009).		114	6.33	2.94

At the same time, more recent publications, despite having fewer total citations due to shorter exposure time, demonstrate increasing influence when considering citation rates per year and normalised impact metrics. These newer contributions tend to focus on emerging themes such as safeguarding, athlete welfare, intersectionality, and governance, indicating a shift in the field from foundational theoretical work toward more applied, policy-relevant research.

While citation-based metrics provide a useful indicator of scholarly influence, they do not fully capture the substantive contributions of individual studies. To address this, it is important to briefly contextualise the most cited works in relation to their thematic contributions. Many of the highly cited articles identified in this study have played a foundational role in shaping key areas of research on discrimination against women in sport. For example, early contributions have critically examined gendered power relations, media representation, and identity construction, while later studies have expanded the field by addressing safeguarding, athlete welfare, and institutional accountability.

This qualitative perspective helps to explain why certain works have achieved high citation impact, as they not only introduced influential theoretical frameworks but also addressed pressing sociocultural and policy issues within sport. Although a full qualitative content analysis is beyond the scope of this study, integrating this interpretive perspective enhances the understanding of how citation patterns relate to the intellectual development of the field.

Table 5 provides a temporal perspective on the most influential contributions in the field, highlighting how research priorities have evolved over time. Early work (1995–2004) primarily established foundational frameworks addressing gender discrimination, harassment, and media stereotyping. The subsequent decade (2005–2014) expanded the scope toward sociocultural and identity-based analyses, particularly in relation to media and representation.

**Table 5:** The most influential articles by decade in women’s discrimination in sport.

Decade	Representative Highly Cited Articles	Key Focus/Contribution
1995–2004	Brackenridge (1997); Young (1997); Koivula (1999)	Foundational work on gender discrimination, harassment, and media stereotyping in sport
2005–2014	Cooky and McDonald (2005); Daniels (2009); Antunovic and Hardin (2015)	Expansion into media representation, identity, and sociocultural analysis
2015–2019	Bruce (2016); Cooky et al. (2021); Thorpe (2017)	Growth of media discourse, digital representation, and feminist critique
2020–2026	Kerr et al. (2020); Bekker and Posbergh (2022); Næss (2026)	Focus on safeguarding, athlete welfare, governance, and inclusion frameworks

From 2015 onwards, the field experienced significant growth, with increased emphasis on digital media, feminist critique, and structural inequalities. More recent research (2020–2026) reflects a clear shift toward policy-oriented and applied domains, including safeguarding, athlete welfare, governance, and inclusion.

This temporal progression demonstrates that while early studies continue to accumulate citations as foundational references, newer research is actively reshaping the field by addressing contemporary challenges and institutional reforms in women’s sport.

The citation analysis reveals that the work that has the most overall citations has been cited 276 times. Thus, it seems to be a seminal work on the conceptualisation of female athletic identity and cultural representation in sport. It has been published earlier; however, its sustained citation rate of 11.5 per year provides evidence of its theoretical timelessness. Among more recent publications, (Bruce, 2016) demonstrates exceptional influence, recording 257 total citations and the highest citations per year (23.36), along with a markedly elevated normalised citation score (8.51). This indicates a strong contemporary impact, particularly in advancing critical discourse on media representation and gender framing in sport. Similarly, Thorpe (2017) and Litchfield (2018) exhibit high normalised citation values (5.62 and 5.90, respectively), reflecting their prominence in shaping intersectional and sociocultural perspectives on women’s experiences in sport.

Classic empirical and theoretical contributions are also well represented. Works by (Amorose & Horn, 2000; Brackenridge, 1997) remain highly influential, with total citations exceeding 200 and 180, respectively. These studies laid foundational frameworks for understanding gendered power relations, leadership dynamics, and safeguarding concerns in sports contexts. The articles in (Harrison & Fredrickson, 2003; Koivula, 1999) further underscore the centrality of media discourse, gender norms, and identity construction in the scholarly examination of discrimination against women in sport. The citation patterns indicate that the most influential literature spans both foundational works and high-impact contemporary studies, collectively addressing key dimensions such as gender inequality in sports governance, leadership barriers, media representation, harassment and abuse, and structural exclusion. In this study, the terms “impactful” and “influential” refer to articles with high citation performance, as measured by total citations (TC), citations per year (TCPY), and normalised citation impact. These metrics are used as indicators of scholarly influence within the field. The strong citation performance of these articles confirms their pivotal role in shaping the intellectual trajectory of research on discrimination against women in sport and guiding subsequent interdisciplinary inquiry.

### 3.5. Most Prolific and Impactful Authors

The analysis of the most prolific and impactful authors is included to identify the key contributors shaping the intellectual structure of research on discrimination against women in sport and to align with this study’s objective of mapping the field’s knowledge base and scholarly leadership. The most prolific and impactful authors contributing to this domain are presented in Table 6, ranked using author-level productivity and citation-based impact indicators, including the h-index, g-index, m-index, total citations (TC), number of publications (NP), and the year of first publication in the dataset (PY_start). Fasting K. is the most productive and influential in the long term, with the largest h-index (8) and g-index (8), eight publications, and 486 total citations since 2002. Conversely, Brackenridge C. exhibits the most significant overall citation influence, with the most total citations (TC = 535), though with fewer publications (NP = 6), indicating the foundation and well-cited evidence of her work on safeguarding, abuse, and gender power relationships in sport. Among the other core authors, Weaving C. ranks third, with NP = 5, indicating that this author has been active since 2009 but has relatively low citation accrual (TC = 53). Bruce T. is a high-impact author with relatively few publications (NP = 3) and very high citation performance (TC = 463), which indicates that a few important articles have disproportionately shaped the academic community, especially in media presentation and gender framing in sport. The body image, sexualisation, and media effects on youth, in particular, are central to the literature, and Daniels E.A. also shows significant effects (TC = 263; NP = 3).

The recent contributors have good momentum, as measured by the m-index, which is the citation impact of the academic career length in the dataset. Liu W. has the most extensive m-index (0.50), starting with publications in 2021, indicating that the index accrued citations very quickly. High m-index values (0.43 each) have also been observed in Kerr G. and McGannon K.R. since 2020, suggesting a growing impact, especially on the welfare of athletes, institutional accountability, and sociocultural aspects of discrimination. Altogether, the author impact profile indicates a mix of well-established foundational researchers (e.g., Fasting and Brackenridge) and high-growth modern researchers (e.g., Kerr, McGannon, and Liu), who influence the development of research on discrimination against women in sport.

**Table 6:** The most prolific and impactful authors contributing to research on discrimination against women in sport.

Rank	Author	h-Index	g-Index	m-Index	TC	NP	PY_Start
1	Fasting K.	8	8	0.32	486	8	2002
2	Brackenridge C.	6	6	0.20	535	6	1997
3	Weaving C.	4	5	0.22	53	5	2009
4	Bruce T.	3	3	0.10	463	3	1998
5	Chroni S.A.	3	3	0.19	102	3	2011
6	Daniels E.A.	3	3	0.17	263	3	2009
7	Kerr G.	3	3	0.43	134	3	2020
8	Litchfield C.	3	3	0.25	142	3	2015
9	Liu W.	3	3	0.50	38	3	2021
10	Mcgannon K.R.	3	3	0.43	68	3	2020

### 3.6. Country and Institutional Collaboration Patterns

**Figure 3:**
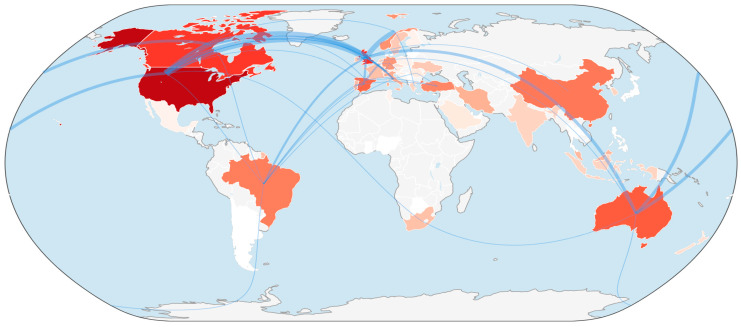
Country collaboration map.

The global and institutional collaboration networks in research on discrimination against women in sport reveal important patterns in the distribution and production of knowledge within the field. The country collaboration map (Figure 3) illustrates that research activity is highly concentrated in a few leading nations, with the United States, the United Kingdom, Canada, and Australia emerging as dominant contributors. These countries demonstrate strong publication output and extensive international co-authorship networks, reflecting well-established research infrastructure, policy engagement, and academic investment in gender equity in sport. European countries such as Norway, Sweden, Spain, and Germany also show active participation, contributing to dense cross-continental collaboration patterns.

**Figure 4:**
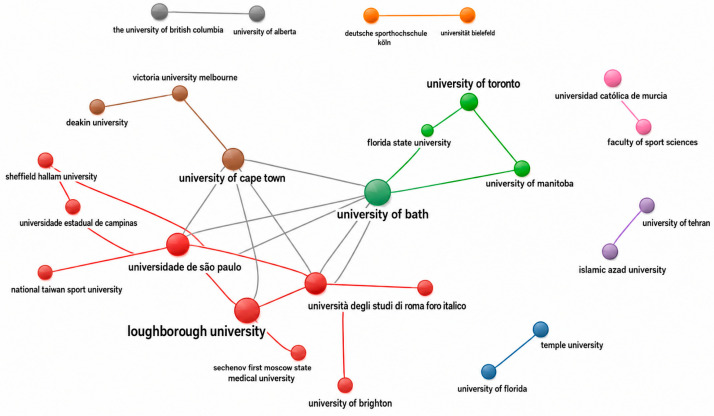
Institutional collaboration network in research on discrimination against women in sport. Node size represents the number of publications contributed by each institution, while the links indicate co-authorship collaborations between institutions.

At the institutional level (Figure 4), key universities—including Loughborough University, University of Bath, University of Brighton, University of Cape Town, and Universidad de São Paulo—serve as central nodes in the global research network. These institutions not only contribute significantly to publication output but also facilitate both regional and international collaborations, indicating their role in shaping the intellectual and empirical development of the field.

Importantly, the combined country and institutional patterns highlight a geographical imbalance in knowledge production, with relatively limited contributions from many regions in Africa, Asia, and South America. This concentration suggests that the research landscape is predominantly influenced by Global North institutions, which may limit the diversity of perspectives and contextual applicability of findings. At the same time, the presence of emerging collaborations from institutions in the Global South indicates a gradual expansion of the research network. While the United States, the United Kingdom, Canada, and Australia emerge as leading contributors, this pattern should be interpreted with caution. The predominance of these countries may partly reflect the reliance on Scopus-indexed, English-language publications, which tend to favour research produced within Anglophone academic contexts. As such, the observed distribution may indicate not solely differences in research quality but also the influence of language, editorial practices, and international publication networks that shape visibility and citation patterns in global scholarship.

**Figure 5:**
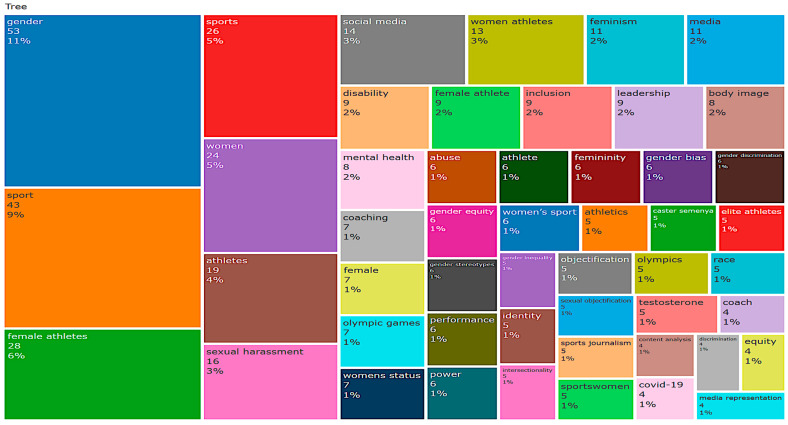
Word tree map.

Figure 5 presents a word tree map of authors’ keywords, where the size of each block represents the relative frequency of terms within the dataset. The prominence of keywords such as gender, sport, and women indicates that the literature is strongly centred on gender-based analysis within sports contexts. Additional frequently occurring terms, including female athletes, media, sexual harassment, and leadership, highlight the multidimensional nature of discrimination, encompassing structural, sociocultural, and representational dimensions.

The distribution of keywords further reveals emerging areas of research, such as inclusion, intersectionality, mental health, and social media, indicating a shift toward more nuanced and interdisciplinary approaches. Overall, the word tree map provides a visual overview of thematic concentration in the field and supports the identification of dominant and evolving research priorities in studies on discrimination against women in sport.

**Figure 6:**
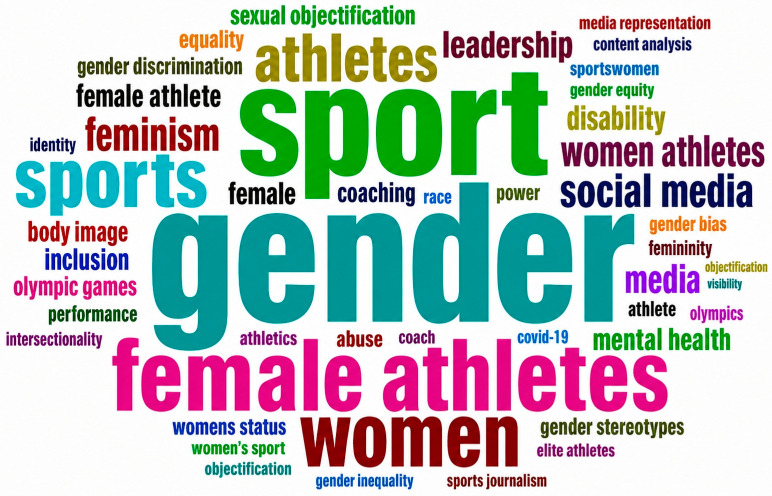
Word cloud.

Figure 6 presents the word cloud of authors’ keywords, where the size of each term reflects its frequency within the dataset. The dominance of terms such as gender, sport, female athletes, and women indicates that the literature is primarily centred on gender-based analysis within sports contexts. The presence of keywords such as media, sexual harassment, leadership, and body image highlights the multidimensional nature of discrimination, encompassing structural, sociocultural, and psychological dimensions. This figure supports the identification of dominant thematic areas in the field and complements the subsequent network-based analysis.

Overall, these collaboration patterns reflect a field that is both globally connected and structurally uneven, where established research hubs drive knowledge production, while opportunities remain for broader inclusion and diversification. This underscores the need for increased participation from underrepresented regions to develop a more comprehensive and context-sensitive understanding of gender discrimination in sport.

### 3.7. Author Performance

The primary objective of this analysis is to identify scholars who have made pivotal and sustained contributions to the research domain of discrimination against women in sport. Table 5 ranks the most significant authors based on fractionalised publication counts and total publication counts. This study prioritises fractionalised counting, as it provides a more accurate representation of individual scholarly contribution by accounting for co-authorship patterns and collaboration intensity and is therefore considered more robust than simple publication counts.

Fractionalised metrics indicate that Fasting K. is the most powerful figure in the field, as they shows long-term productivity and intellectual leadership in various collaborative projects. Brackenridge C. is the next one, and they have exerted significant scholarly influence through their foundational contributions on safeguarding, abuse, and gender power relations in sport. Other prominent authors include Weaving C., Bruce T., and Daniels E.A., whose work has been influential in shaping modern-day discussion of governance, media representation, body image, and structural inequality in women’s sport.

Scientifically, these authors are the intellectual nucleus of the science-mapping field, often the co-authors and citation groups. Not only have their works established the conceptual themes of discrimination against women in sport, but they have also influenced the development of the research agenda toward more critical, intersectional, and policy-relevant questions. The fact that a relatively small group of scholars is dominant implies that although the discipline is growing at an alarming pace, it remains under the influence of a small number of influential voices that shape the direction of theoretical development and applied research.

### 3.8. Science Mapping

**Figure 7:**
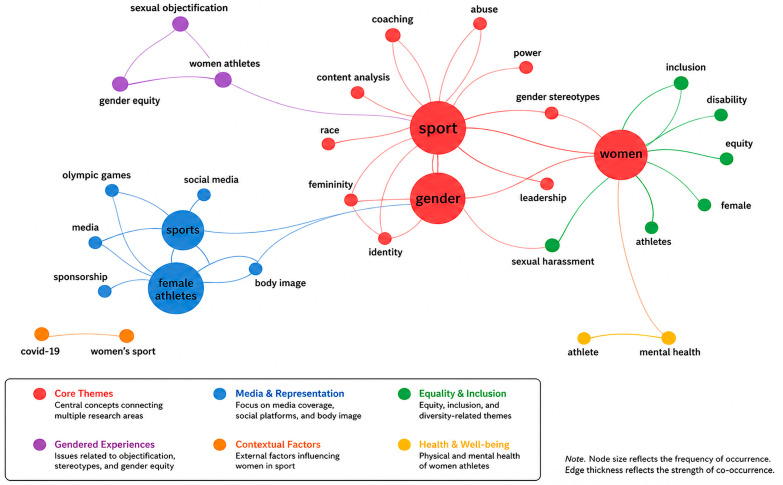
Co-occurrence map.

**Figure 8:**
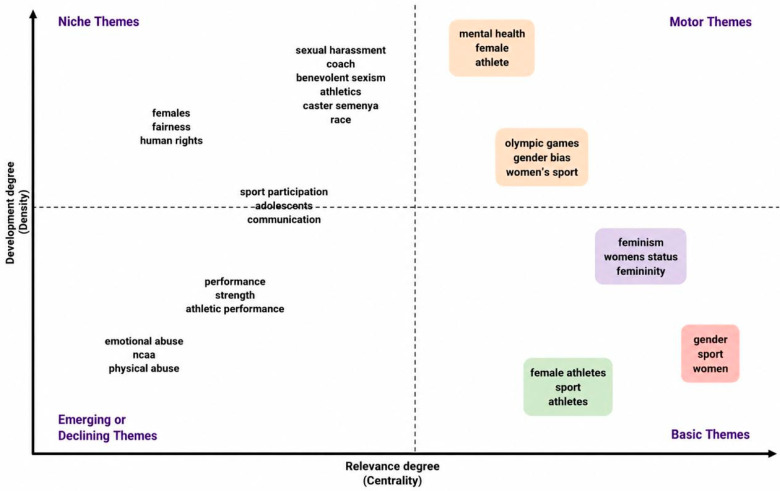
Thematic map.

**Figure 9:**
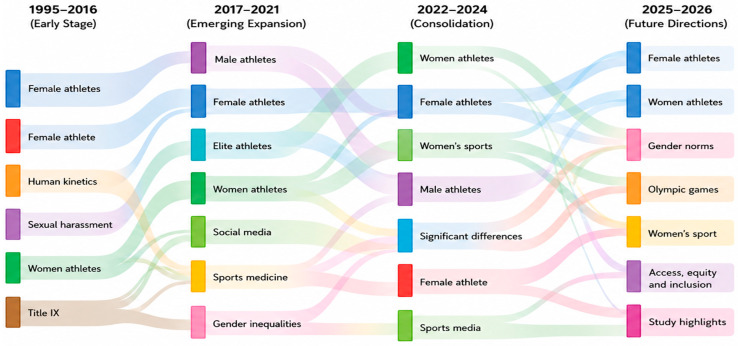
Thematic evolution map of research on women’s discrimination in sport (1995–2026).

This study examines the level of research indexed in Scopus on the topic of discrimination against women in sport to assess the current state of the body of knowledge and identify emerging scholarly interests. This was accomplished using several science-mapping methods obtained from the bibliometric analysis (BA) toolkit. In this respect, the science-mapping section is separated into two mutually complementary parts. The former focuses on the thematic concentration, aiming to frame the current research themes and the field’s intellectual structure. It is achieved through the analysis of the author keyword co-occurrence network (Figure 7), the thematic map (Figure 8), and the thematic evolution map (Figure 9), which collectively reveal the major conceptual clusters and the relationships between them in the study of the problem of discrimination against women in sport.

**Figure 10:**
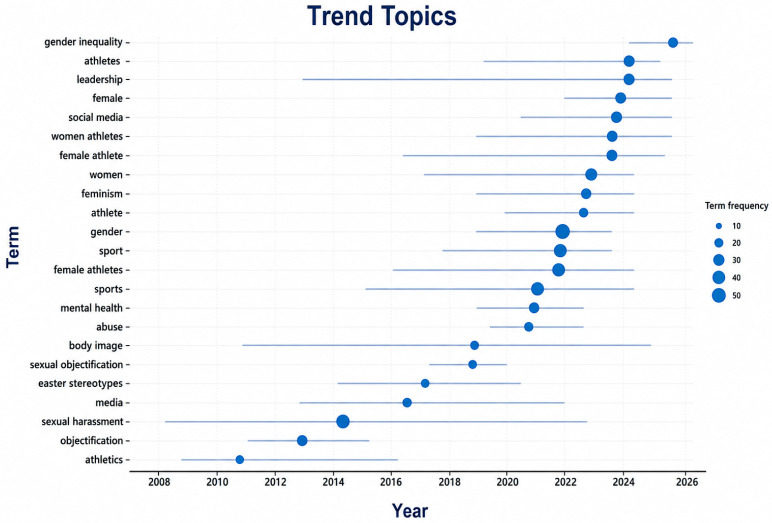
Trend topics.

**Figure 11:**
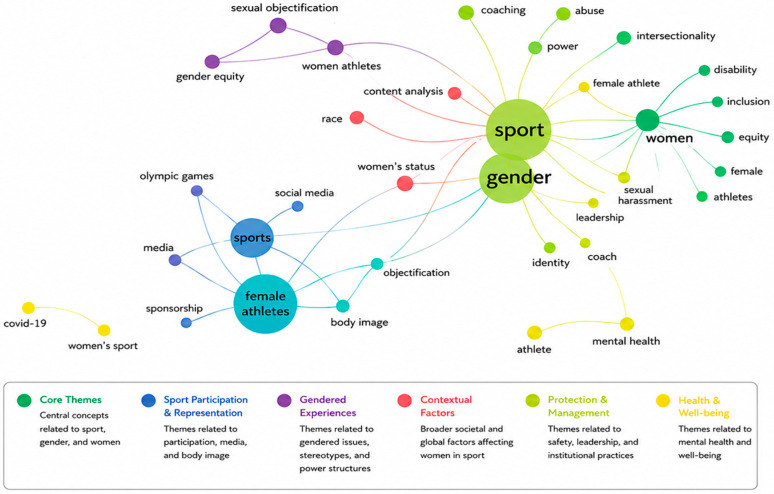
Keyword overlay visualisation.

**Figure 12:**
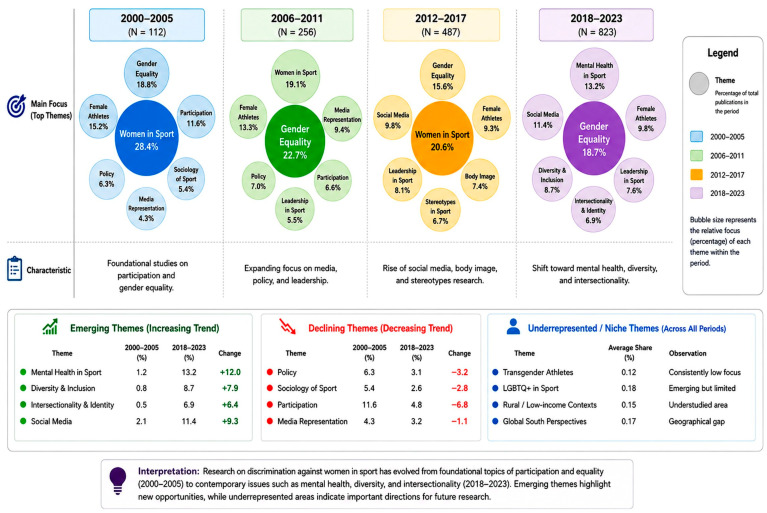
Emerging and Underrepresented Themes in Research on Discrimination Against Women in Sport.

The second element deals with the evolution of the theme and future research directions. This element uses Figure 10 = trend topics, Figure 11 = keyword overlay, and the keyword overlay (Figure 12) to examine changes in the research focus over time and identify new and underrepresented themes. Altogether, these studies provide a prospective view of how research agendas on discrimination against women in sport will develop.

### 3.9. Thematic Concentration

The thematic concentration of research on discrimination against women in sport, as illustrated in Figure 5 and Figure 6, is represented through a word tree map and a word cloud, where the size of each element corresponds to the relative frequency of authors’ keywords within the dataset. The analysis reveals that *gender* is the most dominant theme, accounting for approximately 11% of all keyword occurrences, highlighting its central role in structuring scholarly discourse in this field.

This is followed by *sport* (9%) and *women* (5%), which reflect the broader institutional context and population focus of gender-related research in sport. The prominence of these terms underscores the sustained emphasis on women-centred analyses within sports systems. Keywords directly associated with athlete identity, such as *female athletes* (6%) and *women athletes* (3%), further indicate the continued focus on the lived experiences and representation of women in sport.

Additional themes related to power, representation, and safety emerge through keywords such as *sexual harassment* (3%), *social media* (3%), *media* (2%), *body image* (2%), and *leadership* (2%), highlighting the multidimensional nature of discrimination across structural, sociocultural, and psychological domains. Emerging and cross-cutting areas—including *disability* (2%), *inclusion* (2%), and *feminism* (2%)—indicate a growing shift toward more inclusive and intersectional perspectives.

Furthermore, a range of lower-frequency keywords, each contributing approximately 1%, such as *mental health*, *gender bias*, *race*, *intersectionality*, *objectification*, *safeguarding*, and *coaching*, reflect increasing scholarly attention to nuanced, context-specific, and policy-relevant aspects of discrimination. Collectively, these patterns demonstrate the evolution of the field from descriptive gender-based analyses toward more complex, interdisciplinary, and critical approaches addressing equity, representation, and athlete welfare in sport.

Figure 7 illustrates the keyword co-occurrence network, providing insight into the intellectual structure of research on discrimination against women in sport. The central cluster, dominated by terms such as *gender*, *sport*, and *athletes*, reflects the core focus on structural inequalities. Additional clusters reveal interconnected thematic areas, including media representation and objectification, inclusion and equity, and feminist perspectives. These clusters demonstrate how research has evolved from descriptive analyses toward more critical, intersectional, and policy-oriented approaches, thereby reinforcing this study’s objective of mapping the conceptual and thematic development of the field. The red cluster, which is located in the centre of the map, is the nucleus of the literature on the theme. The keywords sport and gender dominate this cluster (Kascelan et al., 2025), along with words closely related to them, including: women, athletes, sexual harassment, leadership, identity, coaching, power, and gender stereotypes (Marshall et al., 2025). The centrality and density of this cluster suggest that gender-based discrimination in sport is studied primarily through the lens of structural inequalities, governance, power relations, and athlete protection.

The blue cluster focuses on media and representation, and its key keywords are sports, female athletes, media, social media, objectification, and body image (Liang, 2011). This cluster demonstrates a clear but closely related body of research investigating the impact of traditional and digital media on the sexualisation, visibility, and social construction of female athletes, which supports or disrupts discriminatory practices. The green cluster represents an inclusion and equity-related theme, with such keywords as female athlete, inclusion, equity, disability, and sexual harassment (Culver et al., 2019; Patel, 2021). This cluster highlights policy-oriented and rights-based perspectives, which are indicators of increased academic interest in inclusive sports settings and in gender and disability interactions.

The purple cluster aligns with theoretical and feminist viewpoints, focusing on keywords such as feminism, gender equity, sexual objectification, female athletes, and abuse (Gillard et al., 2025). This group forms the conceptual basis of the field, which relies on feminist theory and critical sociology to explain gendered power relations in sport. Smaller peripheral clusters, such as the orange cluster (mental health and athletes), and isolated nodes, such as COVID-19 and the Olympic Games, represent new or context-specific research directions that are not as integrated but have become increasingly relevant in recent years (Bowes et al., 2022).

Figure 8 presents the thematic map of authors’ keywords related to the problem of discrimination against women in sport organised according to two measures: the degree of relevance (centrality) is measured along the horizontal axis and the degree of development (density) along the vertical axis. This map divides themes into four different quadrants, basic themes, motor themes, niche themes, and emerging or declining themes, and thus indicates the conceptual salience of research topics in the field as well as the levels of scholarly development of these topics. Beyond descriptive classification, the thematic map provides critical insight into the structural dynamics and developmental trajectory of the field. The positioning of gender, sport, and athletes as central yet moderately developed themes confirms their role as foundational pillars that connect multiple research streams. The presence of women’s sport, gender prejudice, and mental health within the motor themes quadrant indicates that current research is increasingly driven by issues that are both conceptually central and empirically well-developed, reflecting a shift toward applied and policy-relevant inquiry.

In contrast, niche themes such as sexual harassment, race, and human rights—while highly developed—remain less integrated into the broader research framework, suggesting opportunities for greater conceptual integration. Emerging themes, including emotional abuse, athletic performance, and adolescents, highlight underexplored areas that may shape future research directions.

Collectively, these patterns demonstrate that the field is transitioning from a focus on documenting gender disparities toward a more complex and integrated research agenda addressing governance, safeguarding, intersectionality, and athlete well-being. This reinforces this study’s aim of mapping not only the current structure but also the evolving trajectory of research on discrimination against women in sport.

The quadrant of fundamental themes situated in the lower-right section includes topics that are most centralised but still moderately developed. Keywords dominant in this category include gender, sports, athletes, and female athletes. The fact that gender is a central node implies that it serves as the cornerstone connecting various streams of research on discrimination in sport. These themes are the conceptual pillars of the field, providing the necessary theoretical and empirical foundations across several subdomains. The quadrant of motor themes in the upper-right part contains highly central and well-developed topics: women’s sport, gender prejudice, the Olympic Games, and mental health. The themes are internally coherent and relevant, suggesting they help drive current research priorities. They are positioned as mature inquiry lines that are actively used to formulate policy discourses, governance reforms, and applied interventions to reduce discrimination in sport.

The quadrant of niche themes, located in the upper-left, comprises internally well-developed and specific topics such as sexual harassment, benevolent sexism, race, Caster Semenya, human rights, and fairness. Despite the high density of concepts in these themes, their lower centrality means that they remain more specialised, focusing on specific contexts, case studies, or theoretical debates within the larger area. The quadrant of emerging or declining themes, located in the lower-left, includes emotional abuse, physical abuse, athletic performance, strength, NCAA, and adolescents. The centrality and density of these themes are relatively low, suggesting a research path that is only distilling a topic that has decreased over time. The thematic map, therefore, indicates a systematic and dynamic knowledge domain where the conceptual foundations of gender underpin the field, motor themes drive current research, and niche themes enrich specialised fields. Emerging themes offer potential for future scholarly investigation. It is this structure that emphasises the development of research on female discrimination in sport and the opportunities to incorporate new problems into the existing theoretical frameworks.

Figure 9 illustrates the thematic evolution of research on discrimination against women in sport across different time periods, revealing a clear shift in focus from general descriptive themes to more specific discrimination-related issues. In the early phase (1995–2016), research primarily centred on broad categories such as female athletes, women athletes, and participation, reflecting an initial effort to document gender disparities in sport.

In the subsequent period (2017–2021), there is a noticeable emergence of themes related to media representation, sexual objectification, and gender bias, indicating a transition toward examining the sociocultural mechanisms that sustain discrimination.

In the most recent phase (2022–2026), the thematic evolution highlights a stronger focus on structural and institutional dimensions of discrimination, including leadership inequality, governance, harassment and abuse, safeguarding, and intersectionality.

Themes of research during the first period (1995–2016) were mainly foundational and descriptive. Terms such as female athletes, sexual harassment, human kinetics, and women athletes were dominant, suggesting that there were early attempts to document gender-based differences, athlete identity, and safety issues in sport (Brackenridge, 1997; Hargreaves, 2002; Messner, 2002; S. Shaw & Frisby, 2006). The stage is characterised by the definition of central ideas concerning the role of women in sport and the lack thereof and by the creation of feminist and sociological theories of sports institutions.

The third phase (2022–2024) represents a shift to more specific, contextual themes. Though the prominence of female athletes and women athletes does not disappear, new topics such as women’s sport, sports media, and comparative gender differences are emerging, and they reflect the increase in the emphasis on institutional representation, evidence-based assessment, and inequalities in the area of governance in sport (Bruce, 1998; Cooky et al., 2021). This change suggests the broadening of academic approaches, including the reflection on media power, health conditions, and structural injustices, alongside traditional gender-related discussions (Culvin & Bowes, 2021; Kerr et al., 2020).

The thematic evolution map in the most recent period (2025–2026) highlights the emergence of prospective, policy-related issues. Keywords such as *gender norms*, *women’s sport*, *Olympic Games*, *access*, and *inclusion* dominate this phase, reflecting growing scholarly interest in normative frameworks, equity-driven policy reforms, safeguarding systems, and inclusive sports governance (Mountjoy et al., 2016; Piggott & Pike, 2020). These thematic trends are signs that the field is maturing to action research that informs governance, regulation, and social change.

Figure 10 illustrates the temporal distribution of key research topics, providing deeper insights into the evolving focus of scholarship in this field. While earlier research emphasised general themes such as *female athletes* and *participation*, more recent years show a marked increase in topics directly related to discrimination and inequality, including *gender inequality*, *leadership exclusion*, *media representation*, *harassment*, and *abuse*, and temporal development of research themes that are relevant to women, gender, and sport between 2008 and 2024.

The growing prominence of themes such as *intersectionality*, *mental health*, and *inclusion* further reflects a shift toward more nuanced, interdisciplinary, and policy-oriented research approaches. This progression highlights an increasing scholarly emphasis on addressing structural inequalities, institutional accountability, and athlete welfare, thereby reinforcing this study’s objective of mapping the evolving intellectual and thematic landscape of research on discrimination against women in sport.

The horizontal axis shows the year of publication, and the vertical axis lists keywords of the most dominant authors. The size of the bubbles is directly proportional to the frequency of the term, thus indicating the relative popularity and academic focus on particular issues of a topic over time (Aria & Cuccurullo, 2017). In the early phase (2008–2012), research attention was comparatively limited and concentrated on foundational issues such as athletics, objectification, and sexual harassment. These themes reflect earlier scholarly attempts to document gender-based discrimination, abuse, and representational inequalities in sport, largely in a descriptive and problem-identification mode (Messner, 2002; Mountjoy et al., 2016). During this period, the literature primarily focused on exposing overt forms of sexism and maltreatment experienced by women athletes.

A significant increase in thematic diversity characterises the transition period (2013–2017). Media, sexual objectification, body image, and female athlete(s) become more dominant and indicate that the scope of discrimination has changed to a sociocultural and symbolic level. This period coincides with the increased academic attention paid to media representations, gendered forms, and psychosocial consequences of visibility and sexualisation in sport (Bruce, 1998; Cooky et al., 2021; Daniels, 2009). During this time, studies began moving away from documentation and toward a critical examination of power relations and the representation of cultures. Another significant increase in the number and scope of gender-related topics characterises the consolidation and development stage (2018–2021). Keywords include gender, sports, female athletes, mental health, and abuse. This pattern indicates the incorporation of athlete welfare, psychological health, and protection issues into gender-and-sport studies, in tandem with worldwide policy discussions and institutional responses to athlete abuse (Kerr et al., 2020; Mountjoy et al., 2016). The literature during this stage gradually began to address the health and well-being consequences of discrimination for female athletes.

In the most recent period (2022–2024), the thematic focus becomes more nuanced and policy-oriented. This stage is dominated by high-frequency issues, such as gender inequality, women’s sport, leadership, social media, feminism, and elite athletes. The increase in leadership and gender inequality issues can be interpreted as a shift from identifying disparities to a critical analysis of the mechanisms of structure, organisation, and governance that sustain discrimination in sport (Burton, 2015; Piggott & Pike, 2020). Social media has been highlighted as long-term, underscoring its growing importance in athlete activism, identity formation, and the social discussion around gender equity (Thorpe, 2017).

Figure 11 is a keyword overlay visualisation built on the author keyword co-occurrence data, with a minimum threshold of four occurrences. The frequency of keywords is coded in node size, co-occurrence strength in link strength, and colour in colour gradients, reflecting the average year of publication for each term. The words gender and sport take a central place on the map, highlighting their role in organising the research on the discrimination against women in sport. The terms closely associated with women, athletes, and female athletes highlight the highly athlete-centred nature of the literature. Subnetwork clusters of media, social media, objectification, and body image demonstrate the continued, steadily growing concern with questions of representation and visibility. The keywords related to protection and management, such as sexual harassment, abuse, leadership, and coaching, are becoming more popular, which indicates the transition to institutional responsibility and safety of the athlete. Recent temporal colouring can be identified with the emergence of intersectionality, race, disability, mental health, and inclusion as the themes, which represent the growing intersectional and policy-driven research agenda.

## 4. Discussion

This Scopus-based bibliometric study mapped 397 documents (1995–2026) to clarify the growth trajectory, intellectual structure, and thematic evolution of scholarship on discrimination against women in sport. By combining performance indicators with science mapping (co-word networks, thematic mapping, thematic evolution, trending topics, and overlay visualisation), the findings demonstrate that the field has shifted from early descriptive and feminist critiques to a more policy-relevant, intersectional agenda, addressing leadership inequities, mediated representation, and failures in safeguarding. Importantly, these developments also highlight that gender discrimination in sport is closely linked to athletes’ subjective well-being, as structural inequalities, exclusion, and unsafe environments can negatively influence mental health, motivation, and overall sports experience.

### 4.1. Growth and Consolidation of Scholarship

The publication trend analysis reminds us that there has been an apparent acceleration since the mid-2010s, suggesting that research on discrimination against women in sport is no longer a marginal area but a well-established academic field. This growth aligns with an increase in institutional and societal attention to gender inequalities in sports systems, including governance shortcomings, unequal pay, and politics of visibility in relation to female athletes (Hargreaves, 2002; Messner, 2002). These systemic inequalities are increasingly recognised as key determinants of athlete well-being, as they contribute to psychological stress, reduced motivation, and limited opportunities for personal and professional development. The focus of academic production in specialised and interdisciplinary publications, including *Communication and Sport*, *Women in Sport and Physical Activity*, *Journal of Sport and Social Issues*, *International Review of the Sociology of Sport and Sex Roles*, and others, indicates that the discipline is still based mainly on the sociology of sports and gender studies but increasingly incorporates the perspectives of communication, psychological and ethical methodologies (Bruce, 2016; Burton, 2015; S. Shaw & Frisby, 2006).

Citation analyses also help understand the field’s intellectual development. The most referenced articles include both the classical and the new literature, indicating that classical sociocultural criticism remains at the centre, while the latest articles have a rapid impact on current discussions. For instance, (Bruce, 2016) demonstrates strong contemporary citation velocity, consistent with the expanding importance of gendered media representation and the transition from traditional media critique to digital and platformed sports communication (Toffoletti & Thorpe, 2018). Similarly, (Brackenridge, 1997) remains foundational for safeguarding discourse, underlining that abuse and harassment have long been structural concerns rather than isolated incidents (Mountjoy et al., 2016).

### 4.2. Core Journals and Knowledge Base

The high-profile outlets identified in the data set are central nodes in theory building and empirical consolidation. Feminist and critical sport scholarship has been shaped historically by journals such as *Journal of Sport and Social Issues* and *International Review of the Sociology of Sport*. At the same time, magazines like *Communication and Sport* and *Sex Roles* reiterate the strong role that media systems play in creating as well as challenging gender hierarchies. This allocation supports the view that discrimination in sport exists simultaneously at institutional (allocation of resources, leadership, and policy) and symbolic (visibility, framing, and sexualisation) levels, both of which maintain inequality (Cooky et al., 2015).

### 4.3. Author Impact and Intellectual Leadership

The results at the author level show that the field is influenced by both established scholars and voices that appear quickly. Fasting and Brackenridge are examples of intellectual leadership that have lasted, especially within the gendered relations of power, the sports culture, and the scholarship on protection and abuse—realms that highlight longstanding issues with structural discrimination (Fasting et al., 2014; Kerr et al., 2020). Simultaneously, those authors whose m-index values increased from 2015 to 2021 (e.g., Kerr, McGannon, and Liu) indicate a more recent influence of modern policy discussions, safe sports systems, and athlete welfare. This trend aligns with the field’s maturation toward applied and governance-oriented work that covers reporting mechanisms, institutional accountability, and equity-based frameworks (Mountjoy et al., 2016).

### 4.4. Thematic Concentration

In the word tree, word cloud, co-occurrence network, and thematic map, the conceptual centre is kept in place by gender–sport–women–athletes, as a way of ensuring that the issue of discrimination is analysed chiefly through the lens of women’s participation and experience in male-normed sport structures (Hargreaves, 2002; Messner, 2002). Notably, the science maps demonstrate several large clusters: Structural and leadership inequities (e.g., gender bias, leadership, coaching, and governance) perpetuate the glass-ceiling effects, informal networks, and lack of involvement in decision-making spaces (Burton, 2015; LaVoi & Dutove, 2012). Symbolic discrimination is an active and dynamic theme reflected in media representation, sexualisation (e.g., media, social media, objectification, and body image), and other forms (Bruce, 2016; Daniels, 2009; Toffoletti & Thorpe, 2018). Safeguarding and abuse (e.g., sexual harassment, abuse, and emotional abuse) indicate a heightened concern for athlete welfare and the system’s failure to prevent and respond to abuse (Hartmann-Tews et al., 2020; Mountjoy et al., 2016). Such experiences of harassment, abuse, and exclusion are strongly associated with negative well-being outcomes, including anxiety, emotional distress, reduced self-esteem, and withdrawal from sports participation. Equity and inclusion (including disability and intersectionality) show a shift toward rights-based approaches and stratified disadvantage (Næss, 2026).

### 4.5. Thematic Evolution and Trending Topics

According to the thematic evolution map, a shift in focus from female athletes and sexual harassment to broader, policy-based issues such as gender norms, inclusion, access, and governance in the Olympic movement occurred. This fact is further supported in trend analyses and overlay visualisations that indicate that scholarship after 2020 is increasingly anticipating the future of mental health, safe sport, abuse, and social media. These trends align with a broader shift toward athlete-focused welfare models and digital-age discrimination systems. This shift clearly reflects the growing recognition that gender discrimination is not only a social and structural issue but also a critical factor influencing athletes’ psychological health and subjective well-being. Interestingly, the rise of Caster Semenya as a thematic signifier is an indication of increased involvement in discrimination at the intersection of gender, race, biology, and governance. Regulatory measures regarding sex testing and qualification have become the most essential human rights discourse in elite sport (Mahomed & Dhai, 2019). These developments are in line with a wider methodological and conceptual shift in intersectionality, which emphasises the heterogeneous character of gender discrimination among female athletes (Ratna, 2011).

### 4.6. Gaps and Future Directions

Despite the field’s maturation, the current results indicate that three gaps persist. To start with, there is a strong geographical imbalance, which manifests in the relative lack of representation of Global South contexts, the inability to generalise the findings, and the lack of diversity in cultural and institutional sports systems. Second, the field remains primarily descriptive and critical, with few intervention studies and limited assessment of policy effectiveness, outcome monitoring, or implementation. Third, while media and safeguarding research are expanding, tighter integration with governance, law, and organisational change research is needed to translate evidence into enforceable practice and measurable impact. The observed overrepresentation of Western countries in the literature must also be understood within the broader context of structural and linguistic inequalities in academic publishing. The underrepresentation of the Global South and, to some extent, non-Anglophone regions within Europe may be influenced by factors such as language barriers, limited access to high-impact publication platforms, and the dominance of English-language journals in major indexing databases (Demeter, 2020).

Therefore, the imbalance identified in this study should not be interpreted solely as a gap in research activity but rather as a reflection of unequal visibility and dissemination of knowledge. Addressing this issue requires greater inclusion of diverse publication sources and increased recognition of research produced in different linguistic and regional contexts (Hadad et al., 2025). Future research should more explicitly examine the relationship between gender discrimination and athlete well-being, particularly through longitudinal and intervention-based studies that assess mental health outcomes, life satisfaction, and sustained sports participation (Mountjoy et al., 2022). Such evidence is essential to developing effective policies that promote both gender equity and well-being in sport.

### 4.7. Implications

The findings of this study have several important implications for the future trajectory of research on discrimination against women in sport. The observed concentration of research output within a limited number of countries primarily in the Global North highlights the influence of regional research capacity, funding structures, and policy prioritisation in shaping the field. Similarly, the dominance of leading institutions and a relatively small group of influential authors suggest that knowledge production is concentrated within established academic networks, which play a critical role in defining research agendas and theoretical orientations.

These patterns indicate that future research must move toward greater geographical and institutional inclusivity, particularly by expanding contributions from underrepresented regions in the Global South. Such diversification is essential to ensuring that research reflects a broader range of cultural, social, and institutional contexts, thereby enhancing the global relevance and applicability of findings.

Furthermore, the thematic evolution of the field from descriptive analyses of gender disparities to more critical and intersectional approaches suggests a growing emphasis on structural inequalities, governance, safeguarding, and athlete welfare. Building on this trajectory, future research should prioritise intervention-based and longitudinal studies that evaluate the effectiveness of policies and programs aimed at reducing discrimination in sport.

In addition, emerging themes such as intersectionality, mental health, and inclusion highlight the need for more nuanced and interdisciplinary approaches that consider the complex interplay of gender with other social identities, including race, class, and disability (Bengoechea et al., 2005). Addressing these dimensions will be essential to developing context-sensitive and policy-relevant frameworks.

The inclusion of recent scholarship further illustrates a shift in research priorities toward more critical and applied domains. Studies by Bruce and Fink demonstrate how media systems continue to shape gendered narratives and reinforce inequalities while also evolving through digital platforms. At the same time, research by Kerr et al. highlights increasing scholarly and policy attention to athlete welfare, abuse prevention, and safeguarding in sports systems. Moreover, McGannon’s work reflects a growing emphasis on qualitative and intersectional methodologies, enabling more nuanced and context-sensitive analyses of discrimination. These developments indicate that the field is moving toward more integrated, interdisciplinary, and policy-relevant research frameworks.

Overall, this study underscores the importance of advancing research that not only documents inequality but also contributes to evidence-based interventions, inclusive governance, and equitable sports systems, thereby shaping a more comprehensive and impactful research agenda in the field of discrimination against women in sport.

### 4.8. Limitations and Future Research Directions

This study has several limitations that should be acknowledged. First, the analysis is based solely on the Scopus database, which, although comprehensive, may not capture all relevant publications indexed in other databases, such as Web of Science or PubMed. Second, the findings are dependent on the selected search terms and keywords, which may influence the scope of the retrieved literature and potentially exclude relevant studies using alternative terminology. Third, bibliometric analysis primarily relies on quantitative indicators such as publication counts and citation metrics, which may not fully capture the depth, quality, or contextual significance of individual studies.

Furthermore, this study is limited by the availability and consistency of metadata, including authors’ keywords and affiliations, which may affect the accuracy of thematic and collaboration analyses. While efforts were made to standardise the data, some inconsistencies may remain.

Future research should consider expanding the scope of analysis by incorporating multiple databases and adopting mixed-method approaches, combining bibliometric techniques with qualitative content analysis to gain deeper insights into the nature of discrimination in sport. There is also a need for more region-specific and context-sensitive studies, particularly in underrepresented areas such as the Global South. Additionally, future studies should focus on intervention-based and longitudinal research designs to evaluate the effectiveness of policies and programs aimed at reducing gender discrimination in sport. Emphasising emerging areas such as intersectionality, digital media influence, athlete welfare, and governance reforms will further advance the field and contribute to more inclusive and equitable sports systems.

## 5. Conclusions

This study provides a comprehensive bibliometric and science-mapping analysis of research on discrimination against women in sport, offering insights into the field’s intellectual structure, thematic evolution, and research trajectory. The conclusions presented here are grounded in multiple quantitative indicators, including publication trends, citation patterns, keyword frequency, thematic evolution, and collaboration networks, which collectively inform the interpretation of broader developments within the field.

The analysis reveals a steady increase in scientific production over time, particularly in recent decades. This trend, supported by the growth in publication output and citation activity, indicates a rising scholarly interest in gender-related issues in sport. Furthermore, the prominence of frequently occurring keywords such as gender, sport, and women, along with the increasing visibility of terms related to media representation, leadership, and harassment, reflects a broadening and deepening of research focus within the field.

The thematic evolution and science-mapping analyses provide further evidence of a shift in research priorities. Early research phases were largely centred on documenting gender disparities and participation patterns. Over time, the increasing co-occurrence of keywords such as intersectionality, governance, inclusion, and safeguarding suggests a transition toward more critical, intersectional, and policy-oriented approaches. These findings indicate that the field is evolving from descriptive analyses toward a more complex examination of structural inequalities, institutional dynamics, and athlete welfare.

The collaboration patterns observed in this study highlight a concentration of research output within countries such as the United States, the United Kingdom, Canada, and Australia, supported by strong institutional networks and international collaboration. However, this pattern should be interpreted with caution. Given the reliance on Scopus-indexed, predominantly English-language publications, the observed dominance may also reflect language and database biases, as well as broader structural inequalities in global academic publishing. The relative underrepresentation of the Global South and non-Anglophone regions suggests that current knowledge production may not fully capture diverse cultural and contextual perspectives.

The analysis of highly cited articles further indicates that foundational studies continue to shape the field, particularly in areas such as gender representation, media discourse, and power relations. At the same time, more recent publications—though having lower total citation counts due to shorter exposure time—demonstrate increasing influence when considering citation rates and emerging themes. The addition of a temporal perspective (e.g., most cited works by decade) reinforces the distinction between foundational and contemporary research trajectories, highlighting the field’s ongoing development.

It is important to note that while bibliometric analysis provides valuable quantitative insights into research patterns, it does not fully capture the substantive and qualitative contributions of individual studies. To address this limitation, the present study integrates selective qualitative interpretation of influential works, linking citation patterns to their thematic and conceptual contributions. However, a more detailed qualitative content analysis remains beyond the scope of this study.

Overall, the findings of this study suggest that research on discrimination against women in sport is undergoing a significant transformation, characterised by increasing attention to intersectionality, governance, safeguarding, and inclusive practices. Future research should build on these developments by incorporating more diverse regional perspectives, interdisciplinary approaches, and intervention-based studies, thereby contributing to a more comprehensive and globally representative understanding of gender discrimination in sport.

## Figures and Tables

**Table 1:** Search terms by keywords and database.

Keywords and Database
Scopus
((women OR female* OR girl* OR “women athlete*” OR “female athlete*” OR “women in sport”) AND (sport* OR athlete* OR “elite sport” OR competition OR training OR “sport organization*” OR federation* OR club*) AND (discriminat* OR inequ* OR sexism OR misogyn* OR “gender bias” OR “gender inequality” OR harassment OR abuse OR “sexual harassment” OR violence OR “gender-based violence” OR “pay gap” OR “equal pay” OR “glass ceiling” OR leadership OR governance OR “access barrier*” OR inclusion OR exclusion OR stereotyping OR “media representation” OR objectification))

**Table 2:** Main variables analysed and categories.

Measures	Items	Analysis
Overview	Main information	RStudio/Bibliometrix/Biblioshiny
	Scientific production per year	RStudio/Bibliometrix/Biblioshiny
	Average citations per year	RStudio/Bibliometrix/Biblioshiny
	Three-field plot diagram	RStudio/Bibliometrix/Biblioshiny
Sources	Most relevant journals	RStudio/Bibliometrix/Biblioshiny
	Bradford’s law	RStudio/Bibliometrix/Biblioshiny
Authors	Most relevant authors	RStudio/Bibliometrix/Biblioshiny
	Lotka’s law	RStudio/Bibliometrix/Biblioshiny
Countries	Corresponding authors’ countries	RStudio/Bibliometrix/Biblioshiny
	Scientific production by country	RStudio/Bibliometrix/Biblioshiny
	Most cited countries	RStudio/Bibliometrix/Biblioshiny
Social Structure	Collaboration map between countries	RStudio/Bibliometrix/Biblioshiny
	Author collaboration network	RStudio/Bibliometrix/Biblioshiny
	Author co-citation network	RStudio/Bibliometrix/Biblioshiny

## Data Availability

The data are provided in the tables, and further datasets are available from the corresponding author upon reasonable request.
